# Psychological and social determinants of physical activity from diagnosis to remission among French cancer patients (PERTINENCE): protocol for a mixed-method study

**DOI:** 10.1186/s12889-019-7368-y

**Published:** 2019-08-06

**Authors:** Aurélie Van Hoye, Yacobou Omorou, Christine Rotonda, Sophie Gendarme, Cyril Tarquinio, Bastien Houtmann, Didier Peiffert, Raffaele Longo, Charles Martin-Krumm

**Affiliations:** 1 University of Lorraine, EA4360 APEMAC, Rue du Jardin Botanique 30, 54600 Villers-les- Nancy, France; 2 Inserm, CIC-1433 Clinical Epidemiology, Nancy University Hospital, Nancy, France; 3 National Clinical Research Platform for Quality of Life in Oncology, Besançon, France; 4 Centre Pierre Janet, 57000 Metz, France; 5 Institut de Cancérologie de Lorraine, 54000 Nancy, France; 6 Service d’oncologie, CHR Metz-Thionville, 57000 Metz, France; 7 Laboratoire de Psychologie de l’Ecole de Psychologues Praticiens de Paris, Paris, France; 8 Institut de Recherche Biomédicale des Armées (IRBA), Brétigny, France; 9 ChartUpon EA 4004 Nanterre Paris Ouest, Nanterre, France

**Keywords:** Physical activity, Cancer, Socio-ecological model, Mixed method

## Abstract

**Background:**

Many effective physical activity (PA) interventions have focused on individual factors or a single theoretical model, limiting our understanding of the determinants of PA practice and their interactions in the cancer trajectory. The present mixed-method study aims to capture social and psychological determinants of PA practice from diagnosis to remission among cancer patients, and to identify key levers for PA practice.

**Methods/design:**

A nested sequential mixed-method design QUAN (QUAL+QUAL) will be used, with qualitative studies embedded in the quantitative study to broaden our understanding of the determinants of PA practice. The design is sequential, since qualitative data on medical staff will be collected before patient inclusion (Phase 1), followed by quantitative patient data collection lasting one year (Phase 2) and a final qualitative data collection one year after inclusion (Phase 3). Phase 1 will be a case study in the two hospitals involved in the study, exploring knowledge of and support for PA practice among medical staff. Through interviews and documental analyses, the PA support dynamic will be evaluated with regard to PA prescription. Phase 2 will be a one-year observational study among 693 cancer patients. Quantitative medical, social, dispositional and psychological data, PA practices and preferences, will be collected at diagnosis, and six months and one year thereafter. Phase 3 will be a retrospective study, evaluating societal and policy factors, as well as unexpected factors playing a role in PA levels and preferences among cancer patients. For this phase thirty patients will be identified six months after inclusion on the basis of their PA profiles. Quantitative data will provide the main dataset, whilst qualitative data will complete the picture, enabling determinants of PA practice and their interactions to be captured throughout the cancer trajectory.

**Discussion:**

The present study aims to identify key levers and typical trajectories for PA practice among cancer patients, adapted to different times in the course of cancer and taking into account “what works”, “for whom”, “where” and “how”. The challenge is the tailoring of PA interventions to patients at different times in their cancer trajectory, and the implication of medical staff support.

**Trial registration:**

Clinical Trial NCT03919149, 18 April 2019. Prospectively registered.

## Background

In France, the number of patients living with cancer has increased in the last decade, due to higher remission rates, as well as larger number of diagnosed cancers [1, 2]. The literature has demonstrated that physical activity (PA) can play an important role in decreasing patient mortality rates and in increasing patient well-being at different times during their cancer [3, 4]. In other words, PA is beneficial in primary, secondary and tertiary cancer prevention [5], and helps decrease the side effects of treatment [6]. Previous work has shown that cancer patients tend to decrease their PA from diagnosis to the initiation of treatment [7] and have difficulty in recovering their initial PA level after treatment [8]. Nevertheless, studies focusing only on a specific time in the cancer trajectory (before, during or after) [9] or using a single theoretical model [10] have limited our understanding of the determinants of PA practice and its interactions, as well as of PA trajectories before, during and after cancer.

Different individual factors have been identified as playing an important role in PA practice. For example, gender, age, education level, socio-professional category, ethnicity, or overweight seem to impact PA levels in the general population [11] and among cancer patients [12]. Variables relating to personal disposition (traits), such as hope or optimism, have positive consequences on cancer [13–15], as they encourage the use of coping strategies, especially with regard to motivation [16], passion [17], and emotional regulation [18]. Moreover, physical activity prescription and social support from medical and paramedical staff seem to impact PA practice [19, 20]. While these variables appear to have an effect on PA practice, they are specific to individuals and to their direct environment, and their interactions have not often been analyzed [21].

Beyond the effect of the disease and its treatment, the socio-ecological approach has identified five groups of factors associated with PA practice: individual factors, interpersonal factors, environmental factors, policy factors and global factors [11]. While the first three concern individuals directly or their direct environment or context [22], the last two are more related to societal and policy determinants [23]. To our knowledge, research has mostly focused on one group of factors, especially the individual level, and very few studies have tried to capture all the different levels [21]. In addition, studies have mostly focused on testing the effectiveness of one theory, without crossing different theoretical models, and without taking both social and dispositional variables into account [24]. To be able to capture both the diversity and the interactions over time across the different factors [25], mixed methods are the most suitable. Indeed, by definition, a mixed method seeks to integrate both quantitative and qualitative data to gain a better understanding of a research problem [26], as each separate dataset is not sufficient to capture the trends and details of a situation [27].

### Study aims

**Fig. 1:**
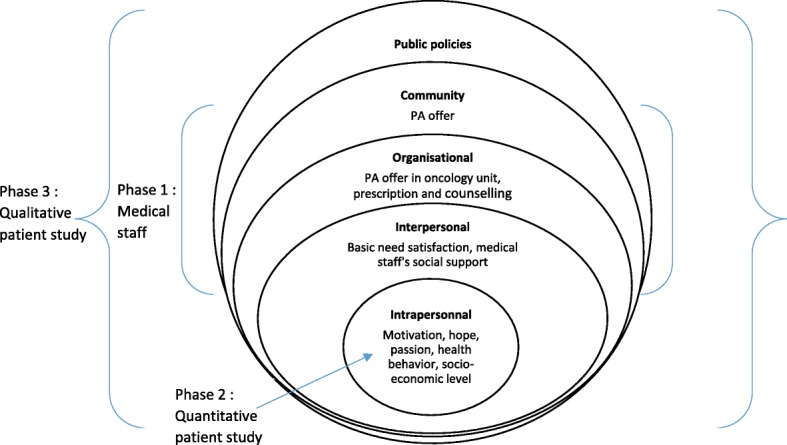
Socio-ecological model used in present protocol

The aim of this mixed-method study is to identify the variables of the socio-ecological model facilitating PA practice, as well as PA levels and preferences (see Fig. 1) at different times during the course of cancer (at diagnosis, during treatment, after treatment). The results from the present work will help to identify key levers of PA practice and their interactions among cancer patients, from diagnosis to remission, in a socio-ecological approach.

The specific objectives are:

1. to evaluate the evolution of PA practices (level, context, preferences) from diagnosis to remission;
2. to understand the interactions and reciprocal effects between sociological, dispositional, psychological and situational variables and PA level, and their evolution from diagnosis to remission;
3. to analyze how policies and the social environment (medical staff) promote PA practice among cancer patient, crossing individual and interpersonal variables.

## Methods/design

The present study is designed as a mixed-method study using a sequential nested design QUAN (QUAL+QUAL) [28, 29]. The sequential design is based on a three-phase data collection process (see Table 1 for details), where qualitative data on medical staff will be collected before patient inclusion, followed by patient quantitative data collection lasting one year and a final qualitative data collection on a sample of patients one year after inclusion. The design will be nested because the three data collections will address different research questions, since the analysis of individual and interpersonal factors will be based on quantitative data and qualitative findings from medical staff, and patient data will complete the socio-ecological model for the social and policy environment. An interview guide will be used in the patient qualitative data collection, and the theoretical patient sampling for phase 3 will be informed by quantitative data from phase 2. The integration of the results will consist in the interpretation and explanation of the quantitative results, also making use of the qualitative results.

**Table 1:** Description of the mixed method design

	Research question	Procedure	Data collection	Data analysis	Expected results	Data integration
Phase 1	What is observed among medical staff in terms of knowledge of and support for PA practice?	Case analysis per hospital	Interviews with health professionals using snowball sampling and document analysis	Content analysis using Nvivo software	Information on PA knowledge and support from medical staff, application of prescription requirements and orientation of patients to PA practice	Complete quantitative data on social support, public policies and organizational factors that encourage PA practice
Phase 2	What are the psychological and social factors influencing PA practice from cancer diagnosis to remission?	Cohort study on 680 patients included at diagnosis (T0; + 6 months; + 1 year)	Self-reported questionnaire	Descriptive, multivariate, multilevel analysis using SPSS, SAS and AMOS	Patient profiles for PA practice, theoretical model of interaction over time between social and psychological variables supporting PA practice	Quantitative data underpins the main data analysis Identification of patient profiles for the phase 3 study
Phase 3	What is the evolution of PA practice, PA preferences and what are the main barriers / facilitators from the time of diagnosis?	Qualitative study	30 patients with specific profiles identified in phase 2	Content analysis using Nvivo software	A list of factors, organized in the socio-ecological model to calibrate interventions	Broadening of the quantitative data and more detailed analysis of interactions with interpersonal and political variables

### Study setting

The present study will take place in the oncology departments of two hospitals in the north-east of France: the Lorraine Cancer Institute (ICL) and the Regional Hospital Center of Metz-Thionville.

### Ethics approval

Verbal informed consent will be obtained from each participant and they will be allowed to withdraw at any time without any consequences. Data will be made anonymous and only the first letter of the first name and surname and a number attributed to each patient will be recorded. This research complies with the Helsinki declaration and is registered with the French National Commission for individual privacy (CNIL) and to the French committee for individual protection (CPP). This research is registered on clinicaltrials.gov (NCT03919149).

### Phase 1: study involving medical staff

This phase is designed as a case-analysis [30], to explore knowledge and support on the part of medical staff (oncologists, nurses, physiotherapists, trainers in adapted physical activity) concerning PA practice among cancer patients.

#### Sampling method

As the study involves two different hospitals, the medical staff will be invited to participate in semi-structured interviews and to identify colleagues that would be willing to answer our questions, thus enabling a snowball sampling procedure [31].

#### Data collection

Semi structured interviews [32] with medical staff will be conducted, using an interview guide, based on the six dimensions of the Guide to good prescribing [33], adapted to physical activity.

#### Data analysis

The interviews will be fully transcribed and the content will be analyzed using both deductive methods (based on the Guide to good prescribing [33]) and inductive methods, where the interview content will be coded in the different dimensions of the model. Data will be interpreted by crossing responses obtained from the medical staff, to see which dimension of the Guide to good prescribing [33] can be applied, and to determine cancer patient trajectories. In addition, between and during the interviews, the means deployed to prescribe or support PA will be collected and analyzed, to enrich the data for each case.

### Phase 2: observational study

**Fig. 2:**
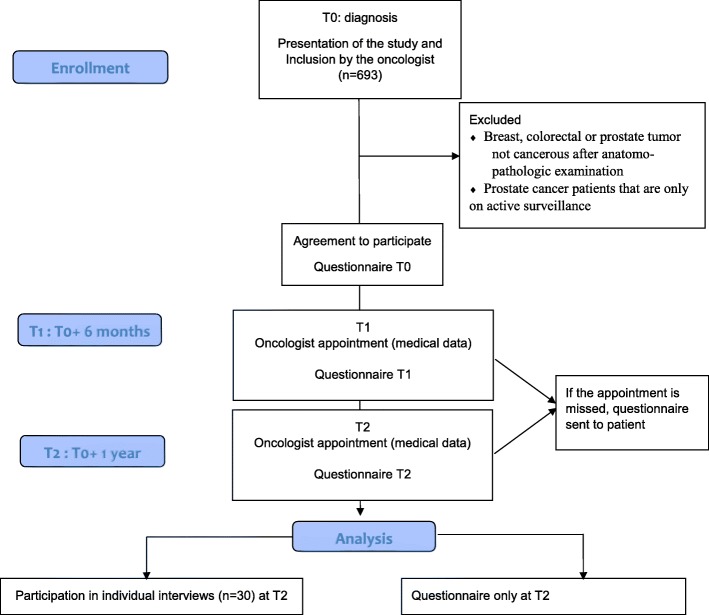
Flow diagram

This phase is an observational study to explore the psychological and social determinants of PA practice among cancer patients from diagnosis to remission. Diagnosis is a moment when patients are particularly sensitive towards behavioral changes, if they are given the appropriate tools [34, 35]. The source population will include breast, colorectal and prostate cancer patients from two hospitals in north-eastern of France, to restrict heterogeneity of the cancers considered (see Fig. 2 for flow diagram).

#### Sample size and sampling method

The principal judgment criterion is the compliance with PA recommendations (i.e. 150 min of at least moderate PA per week). In a previous cohort study [36], 15% of breast cancer patients (*n* = 466) managed to comply with these recommendations. Considering the fact that patients with factors facilitating PA practice are twice as likely to comply with these recommendations (Odds Ratio = 2), with an alpha risk of 5% and a power of 80%, 660 cancer patients will be included (220 per cancer localization). Taking into account a potential loss of 5% of the patients (11 patients per localization), we hope to include and follow a total of 693 patients (231 for each cancer localization). The data collection will last 12 months, reaching 30 patient inclusions per month in each hospital.

#### Eligibility criteria

The inclusion criteria are:

1. Being aged 18 years and older,
2. Newly diagnosed with breast, prostate or colorectal cancer
3. Having been referred to an oncologist before the beginning of any treatment for cancer
4. Being able to provide informed consent to participate in the study

The exclusion criteria are:

1. Presence of a threatening bone lesion
2. Physical, cognitive or linguistic inability to fill in the questionnaire
3. Estimated patient life expectancy under 6 months (oncologist’s clinical opinion)
4. Under custodial sentence or legal protection

Exclusion criteria in the course of the study are:

1. Breast, colorectal or prostate tumor shown to be non-cancerous following anatomo-pathologic examination
2. Prostate cancer patients that are only on active surveillance

#### Scales

The questionnaire will include four main sections and different scales: medical and socio-economic variables, personality traits, situational variables and physical activity. The scales will be chosen on the basis of their validation in French and their suitability to PA and cancer patients, when available.

Medical data will be completed by the clinical research assistant, and will include: patient care plan, actual patient trajectory (treatment completion, frequency, type), symptoms and complementary treatment (fatigue, pain, nutrition).

Sociological variables will include age, gender, education, socio-professional category, professional activity, marital and socio-economic status, and place of residence. An 11-item scale to evaluate precariousness and health inequality (EPICES scores, [37]) will explore all the dimensions of precariousness. Life habits (tobacco, alcohol, substance use) and PA practice before diagnosis will help to identify inter-determinant relationships. Medical data (co-morbidities, fatigue, sleep, pain) will also be collected from patients’ perspective.

Personality traits will be approached via two validated scales: Trait Hope [38] and Trait Optimism [39]. Trait Hope will be assessed using the Dispositional Hope Scale [40], validated in French [38]. The Trait Hope Scale contains four “agency” items, four “pathway” items, and four “filler” items. Respondents will be asked to rate the items on an 8-point Likert scale ranging from 1 “definitely false” to 8 “definitely true”. The Life Orientation Test-Revised (LOT-R, [39]), comprises six items and four fillers, and will be rated on a 5-point Likert scale, ranging from 1 “strongly disagree” to 5 “strongly agree”.

Situational variables will include passion [41], satisfaction of basic needs in sport [42], state-hope [38], self-determined motivation for PA [43], anxiety and depression [44]. The two dimensions of passion (harmonious and obsessive) will be measured on a 12-item scale [41], where answers will be rated on a 7-point Likert, ranging from 1 “totally disagree” to 7 “totally agree”. The basic needs satisfaction (autonomy, competence and relatedness) will be collected using a validated French scale in the sports context [42]. Participants will answer on a 7-point Likert scale, ranging from 1 “totally wrong” to 7 “totally true”. The Adult dispositional Hope scale [38] will be used to explore state-hope. This scale includes 6 items (2 dimensions: operational and motivational components), and will be answered on a 6-point Likert scale, ranging from 1 “totally disagree” to 6 “totally agree”. All six dimensions of self-determined motivation for PA [43] will be explored using an 18-item scale, with a 6-point Likert response scale ranging from 1 “does not correspond at all” to 6 “corresponds totally”. The Health Anxiety Depression Scale, adapted into French [44] will measure anxiety (7 items) and depression (7 items), on a three-point scale.

Physical activity and sedentary behaviors will be measured using the self-reported Global Physical Activity questionnaire [45]. This questionnaire will measure the frequency, intensity, and duration of PA practice, as well as its context (work, mobility or leisure). PA preferences will also be evaluated in terms of type of practice, duration, intensity etc. [46].

#### Data collection

The inclusion of patients will be conducted by oncologists at the first visit to the oncology department (see Fig. 2 for patient flow diagram). The study will be presented verbally to patients that meet the inclusion criteria. Patients will give their verbal consent to participate in the study, and if they agree to participate, the oncologist will contact the clinical research assistant to administer the baseline (T0) survey to the patients. The patients will be allowed to answer the questionnaire directly or to take it home and return it within a month to the research center. The T1 and T2 visits will be integrated into the patients’ clinical follow-up. The clinical research assistant will call a few days before the visit to the oncology department to remind them to plan for a further 20 min during their visit to fill in the questionnaire. Once again, if the patients wish, they will be allowed to take the questionnaire home and return it within a month to the research center. A reminder will be send after 2 weeks. Thirty patients will be contacted at T2 to participate in an interview on a voluntary basis (for phase 3), depending on their PA practice and characteristics.

The questionnaire will be filled in three times by the cancer patients: at diagnosis (T0), six months after diagnosis (T1) and one year (T2) after diagnosis. At each time, patients will respond to the questionnaire on physical activity, preferences and situational variables. At T0, patients will also provide information on medical, socio-economic and personality variables. At T2, patients will also provide information on personality variables, to address the stability of these characteristics among individuals facing such an important event as cancer.

#### Data management

A Microsoft Access-based information system will be developed (Microsoft Access®, 2007). Data will then be stored on a secured server in the CIC-1433 EC (Centre d’Investigation Clinique − 1433 Epidémiologie Clinique) of the teaching hospital. To ensure the quality of the data collection, the independent data monitoring committee will execute a data quality control will be planned.

#### Data analysis

The analyses will be performed using SPSS and SAS software. Basic descriptive statistics, including means, standard deviations and frequencies will be calculated. A comparison of PA levels will be made across groups of patients depending on their hospital site and cancer localization. An identification of PA profiles and trajectories during the study will be conducted using a latent class growth analysis [47]. Relationships between the individual and interpersonal variables of the socio-ecological model will be tested using multilevel structural equation modeling. Profiles of PA practice among cancer patients will also be approached through the qualitative data collection, to identify further societal and policy factors, using Nvivo software.

### Phase 3: retrospective patient study

#### Sampling method

At T2, 30 patients will be recruited to participate in a retrospective personal narrative interview [48] on their PA practice since the cancer diagnosis. The objective will be to analyze societal and political levers for PA practice, as well as to explore the results for the variables collected (especially PA level and preferences) at T0 and T1. These interviews will also help to calibrate future interventions, on the basis of unexpected or non-measured variables detected within phases 1 and 2.

#### Data collection

An adapted retrospective interview procedure [48] will be used to collect PA involvement and various levers, from policies to individual factors, since the cancer diagnosis. As participants will be selected on the basis of their PA profile, retrospective interviews will help to refine their PA preferences, as well as to get a more complete picture of the socio-ecological model. Participants will be asked to identify the type and frequency of PA they engaged in before the diagnosis, at diagnosis, during treatment and after treatment, and which factors played a role in this engagement.

#### Data analysis

Interviews will be fully transcribed, and inductive thematic analyses will be conducted to identify the factors playing a role in PA practices and preferences, using Nvivo software.

### Integration of qualitative and quantitative data

The nested mixed method design will help to address three different questions, but the quantitative data will be the grounding for the integration of the data, whilst the qualitative data will help to provide insight into interpersonal, societal and policy factors influencing PA practice. Phase 2 data will be crossed with phase 1, to identify patient counseling and the PA offer that best supports their PA level at the different times d in the course of the cancer. Phase 2 data will help to identify phase-3 participants, through an analysis of PA trajectories, and the crossing of the two data-sets will provide a picture of the factors to take into account to calibrate a PA intervention for cancer patients based on the socio-ecological model.

## Discussion

Improving PA interventions for patients throughout their cancer trajectory is a major challenge, as the benefits of PA have been demonstrated in numerous studies [3, 4]. The present mixed-method protocol will identify factors at different levels and will cross theoretical models to produce robust data and key levers to calibrate these interventions. The collection of both qualitative and quantitative data will enable better coverage of the plurality of determinants of PA practice [11] and identify PA preferences and the interactions of its determinants at different times, from cancer diagnosis to remission. Studies exploring the habits of both healthcare professionals and patients in PA are rare, and evidence is needed to identify “what works”, “for whom”, “where” and “how” [24]. The study findings will help address this question and support the development of future interventions.

## Data Availability

Not applicable.

## Abbreviations

CIC-1433 EC: Centre d’Investigation Clinique − 1433 Epidémiologie Clinique
CNIL: French National Commission for individual privacy
ICL: Lorraine Cancer Institute
LOT-R: Life Orientation Test-Revised
PA: Physical activity
